# The LIM protein Ajuba/SP1 complex forms a feed forward loop to induce SP1 target genes and promote pancreatic cancer cell proliferation

**DOI:** 10.1186/s13046-019-1203-2

**Published:** 2019-05-17

**Authors:** Bosen Zhang, Liwei Song, Jiali Cai, Lei Li, Hong Xu, Mengying Li, Jiamin Wang, Minmin Shi, Hao Chen, Hao Jia, Zhaoyuan Hou

**Affiliations:** 1 Department of Surgery, Ruijin Hospital, Shanghai Jiaotong University School of Medicine, 197 Ruijin 2nd Road, Shanghai, 200025 China; 2 Hongqiao International Institute of Medicine, Tongren Hospital/Faculty of Basic Medicine, Shanghai Jiaotong University School of Medicine, 280 South Chongqing Road, Shanghai, 200025 China; 3 Department of General Surgery, Shanghai General Hospital, Shanghai Jiaotong University School of Medicine, Shanghai, China; 4 Department of Radiology, Changzheng Hospital, Second Military Medical University, Shanghai, 200003 China; 5 Department of Thoracic Surgery, Lanling People’s Hospital, Lanling County, Linyi, 277700 China; 6 Department of Biochemistry and Molecular Cell Biology, Shanghai Key Laboratory for Tumor Microenvironment and Inflammation, Shanghai Jiaotong University School of Medicine, 280 South Chongqing Road, Shanghai, 200025 China

**Keywords:** Ajuba, SP1, PDAC, Co-activator, FunRich, cBioPortal

## Abstract

**Background:**

The aim of this study is to explore the molecular mechanism of the LIM protein Ajuba and the transcription factor SP1 in the pathogenesis and progression of PDAC. Ajuba is a newly defined transcriptional co-regulator and plays important role in various cancer development, while SP1 is a classic transcription factor, and is closely related with a variety of gene expression and cancer development including PDAC.

**Methods:**

The expression of Ajuba and SP1 in PDAC tissues was detected by immunohistochemistry (IHC), and the correlation between expression level and clinical prognosis of Ajuba and SP1 was extensively analyzed using online tools. The interaction between Ajuba and SP1 was examined by co-immunoprecipitation (co-IP) and GST-pulldown assays. Stable cell lines were established via lentiviral infection, and was examined by qRT-PCR and western blot assays. The effects of Ajuba/SP1 on PDAC cell proliferation were examined using CCK8 and colony formation assays. Luciferase reporter and chromatin immunoprecipitation (ChIP) assays were employed to examine the transcription activity.

**Results:**

The expression level (protein and mRNA) of Ajuba and SP1 was elevated in PDAC tissues and was positively correlated; patients with high Ajuba and SP1 expression had a poor prognosis. Mechanistically, Ajuba binds to the C-terminus of SP1 and functions as a co-activator to enhance SP1 gene expression and promote cell proliferation; the promoter of Ajuba contains functional SP1 responsive elements and Ajuba itself is a target gene of SP1.

**Conclusion:**

Ajuba functions as a co-activator of SP1 to induce its target gene, and that Ajuba itself is a target genes of SP1. Ajuba/SP1 complex could form a feed forward loop to drive SP1 target gene transcription and promote cell proliferation of pancreatic cancer cells. Ajuba and SP1 might be biomarkers for PDAC diagnostics, prognosis and targets for new therapeutics.

## Background

Pancreatic ductal adenocarcinoma (PDAC) is one of the most common lethal malignancies worldwide with the median survival time of 3 to 6 months and the 5-year survival rate of less than 5% [1–3]. The low survival rate of PDAC patients is largely due to the disease’s properties of rapid progression, potent invasiveness and profound resistance to treatments. Additionally, most PDAC patients do not show any symptoms before it can be diagnosed, leading to only 20% of PDAC patients having the chance to undergo radical resection [3]. Thus, identifying biomarkers and new therapeutic targets for early diagnosis and treatment are especially important to reduce the death rate of PDAC patients.

Ajuba belongs to the LIM protein family characterized by the C-terminal tandem LIM motifs, a double zinc finger in structure and a module to mediate protein-protein interaction [4]. Ajuba contains both nuclear localization signal (NLS) and nuclear exporting signal (NES) and can shuttle between nucleus and cytoplasm. This property enables Ajuba a versatile scaffold participating in the assembly of numerous protein complexes to regulate a variety of cellular processes including cell adhesion, cell migration, cell proliferation, apoptosis, mitosis and gene transcription [4–10]. For examples, Ajuba binds the Lats / Wts kinase to negatively regulate the Hippo signaling pathway [7, 8]; Ajuba specifically binds Jak1 to inhibit interferon signalling [9]. In the nucleus, Ajuba acts as an obligate co-repressor of Snail and recruits histone modifying enzymes protein arginine methyltransferase 5 (Prmt5) [10], histone deacetylase (Hdac) and zeste gene enhancer homolog 2 (Ezh2) to directly repress gene expression [11]; moreover, Ajuba can interact with multiple nuclear hormone receptors to either repress or activate gene expression [12–14].

Accumulated knowledge suggests that Ajuba may play important roles in oncogenesis and progression. Indeed, mutations in Ajuba were detected in multiple tumors including esophageal squamous cell carcinoma, skin squamous cell carcinoma and squamous cell carcinoma of the head and neck [15]. However, the exact role of Ajuba in tumor progression remains controversial. A number of literatures reported that Ajuba acts as an oncogene and promotes tumor cell growth and migration, while others showed that Ajuba functions as a tumor suppressor and inhibits tumor cell growth and migration [10, 16].

Specific protein 1(SP1) is a member of the SP / Kruppel-like factor (KLF) transcription factor family, characterized by the presence of three conserved Cys2-His2-type zinc finger DNA binding domains in its C-terminus [17–19]. SP1 binds to the GC box comprising a consensus sequence 5′-(G/T) GGGCGG (G/A) (G/A) (C/T)-3′ via the zinc finger motifs to transactivate gene expression, and about 12,000 SP1 binding sites are found in the human genome [17, 18]. Accordingly, SP1 controls transcription of a large pool of genes involved in regulation of various cellular processes. Numerous literatures have demonstrated that SP1 can promote tumorigenesis, progression and metastasis and its expression level is closely related with tumor invasion and poor prognosis. Epidermal growth factor receptor (EGFR), Insulin-like growth factor 1 receptor (IGF1R), vascular endothelial growth factor (VEGF) and COX-2 are among the list of the best known SP1 target genes that are critical regulators of both normal development and malignancies [20–24].

In this report, we discover that both Ajuba and SP1 are highly expressed in PDAC tissue and their expression is positively correlated; mechanistically, Ajuba functions as a novel co-activator of SP1 to induce EGFR and IGF1R expression and to promote pancreatic cancer cell growth; moreover, Ajuba itself is a direct target gene of SP1. These findings suggest that Ajuba and SP1 might be biomarkers for PDAC diagnostics and targets for new therapeutics.

## Methods

### Immunohistochemistry (IHC) and tissue microarrays (TMA)

IHC staining was performed as previously described [25]. Tissue microarray (TMA) chips (HPan-Ade180Sur-02) were purchased from Shanghai Outdo Biotech Company, which contain 80 cases of paired tumor/ peri-tumor specimens. All specimens were well documented with complete follow-ups for periods from 3 to 7 years. Tumor staging was evaluated according to the TNM Classification of Malignant Tumors. TMA chips were scanned for IHC evaluation. The immunoreactive score (IRS) system [25] was used to evaluate the staining of each spot as follow: negative (−), 0 to 1 point; mild (+), 2 to 3 points; moderate (++), 4 to 8 points; strongly positive (+++), 9 to 12 points. For statistical analyses, IRS score > 4 was regarded as positive expression.

### Co-immunoprecipitation (co-IP) and GST-pulldown assay

Co-IP assays were carried out as described [9, 14]. The antibodies used are as follows: Ajuba (#4897, Cell Signaling Technology), SP1 (#sc-420, SCBT), Flag (Sigma-Aldrich, F7425), Myc (9B11, #2276, SCBT), normal IgG (#2729, Cell Signaling Technology). GST-tagged SP1 and His tagged Ajuba were expressed in BL21 respectively, and purified by Glutathione Sepharose beads (17–0756-01, GE Healthcare) or Ni-beads (17–5318-06, GE Healthcare). For the in vitro binding assays, the purified proteins of GST-SP1 were mixed with His-Ajuba was added into the mixture for 12 h.

### Plasmids

Plasmids PLKO.1-shAjuba, PLKO.1shLuc, Myc-Ajuba and its truncations were previously described [9]. The human SP1 cDNA and its truncations were subcloned into pcDNA3.1-Flag vector or PCDH vector and the Flag-tag was added at the N-terminus of the protein, and were confirmed by DNA sequencing.

### Cell culture, transfections and retroviral infection

HEK-293 T cells and pancreatic cancer cell PanC1 were obtained from the ATCC, and Patu 8988 was purchased from the Type Culture Collection of the Chinese Academy of Sciences (Shanghai, China). The cells were maintained in DMEM supplemented with 10% FBS, 2 mmol/L L-glutamine, and penicillin (50 U/mL)/streptomycin (50 mg/mL) at 37 °C under 5% CO_2_ in a humidified chamber. Transfection was performed using Lipofectamine 2000 (Invitogen) according to the manufacturer’s instruction. The viral supernatants were generated in HEK-293 T cells, and were infected into PanC1 and Patu8988 cells. Puromycin (2.5 μg/mL) was added into the media to generate stable cell lines.

### Cell proliferation and colony formation assay

Cell proliferation assay was performed using cell counting kit 8 (CCK8, Dojindo, Japan) according to the manufacturer’s instruction. To determine cell colony formation efficiency, 1000 cells were plated in 2 mL DMEM and 10% FBS in 6-well plates. After 10 days of growth, the cells were washed in PBS 3 times and dyed with methylrosanilnium chloride solution at a 0.01% concentration for counting.

### Quantitative polymerase chain reaction (qPCR), luciferase and chromatin immunoprecipitation (ChIP)

Total RNA was extracted from cells with TRIzol reagent (Life Sciences, Carlsbad, CA, USA) according to the manufacturer’s instruction. Complementary DNAs were synthesized with 2 μg of total RNA using PrimeScript RT Reagent Kit (Invitrogen). qRT-PCR was performed on a 7500 Fast Realtime PCR system (Applied Biosystem) using SYBR Green agent. The luciferase reporter assays were described previously [9]. The chromatin immunoprecipitation (ChIP) procedure has been described previously [9]. The chromatin was sonicated to fragments about 500 bp, and immunoprecipitation was performed using antibodies against Ajuba and SP1 respectively. Equal amount of normal IgG was used as negative control. The precipitated DNA fragments were extracted and examined by PCR.

### Database analysis and transcription factor prediction

The dysregulated expression genes in pancreatic ductal adenocarcinoma (PDAC) tissues with lower Ajuba expression from TCGA database were extracted by cBioPortal (http://www.cbioportal.org/) [26, 27]. We chose the pancreatic adenocarcinoma (TCGA, provisional), and the alterations of mutation, copy number alteration and expression of mRNA and protein were calculated separately. Transcription factor prediction of the gene list was analyzed using FunRich software [28].

### Statistical analysis

Data shown as mean ± standard deviation (SD) were analyzed by the independent Student t test. The distribution of the IHC scoring results on TMA chips was analyzed by the McNemar test and Pearson’s chi-square test. The correlation between the expression of Ajuba and SP1 in PDAC was analyzed by the Spearman rank correlation coefficient test. The postoperative survival of patients with PDAC was analyzed by Kaplan–Meier estimator and tested by the log-rank. *P* value of < 0.05 was considered statistically significant. Statistical analysis was performed using SPSS 18.0 (SPSS, Inc. Chicago, IL, USA).

## Results

### Expression level of Ajuba predicts poor survival of PDAC patients

**Fig. 1:**
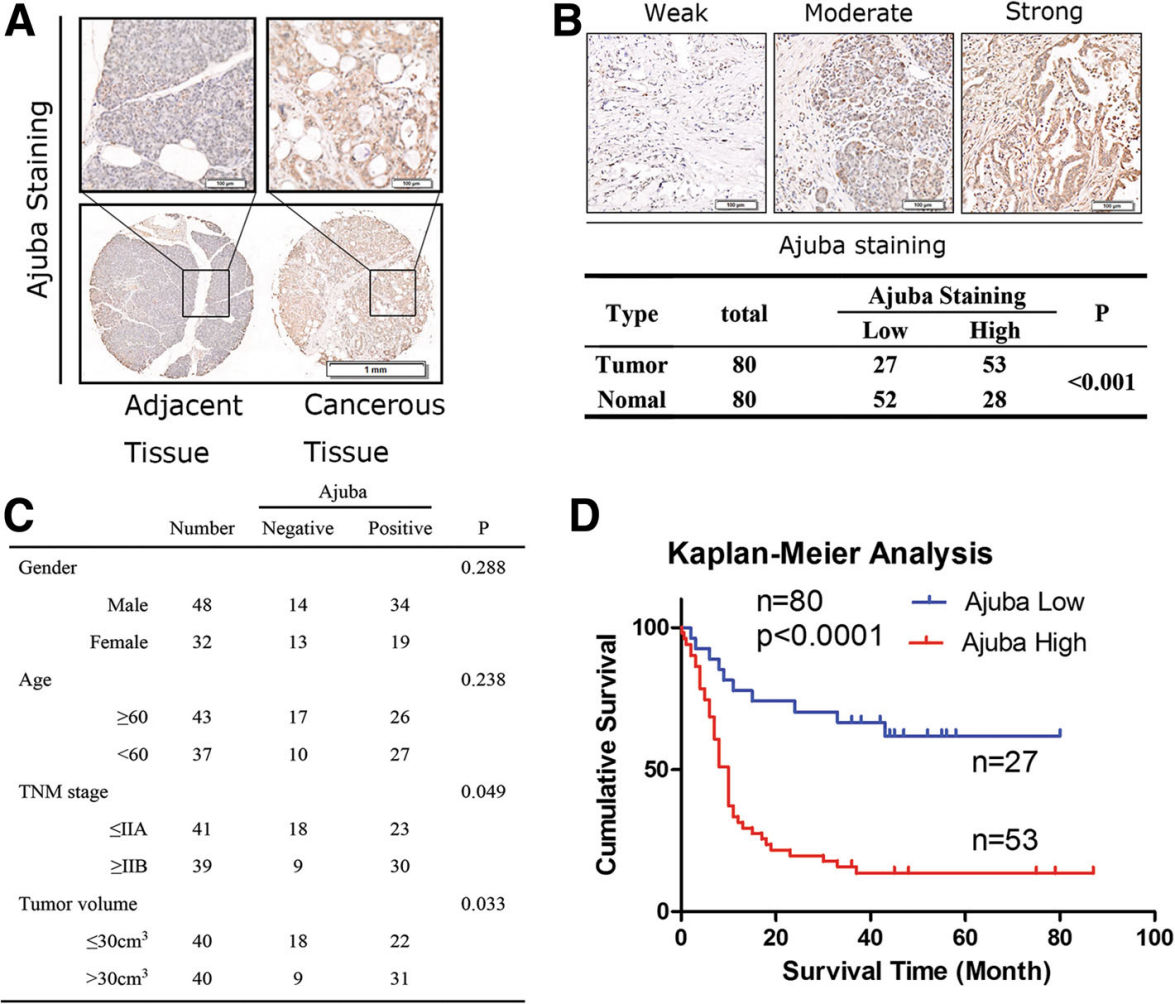
High expression of Ajuba predicts poor prognosis in PDAC. **a** Expression pattern of Ajuba in paired human PDAC specimens (cancerous vs. adjacent tissue) based on intensity and percentage. **b** Representative images of PDAC tissues with weak/moderate/strong Ajuba expression. Statistics of the IHC staining of 80 cases of the PDAC specimens on TMA chips. Only IHC scores ≥4 point (++) was considered as high. Bar: 20 μm. **c** High level of Ajuba is positively associated with TNM stage and tumor volumes. **d** Survival analysis of patients with PDAC by Kaplan–Meier plots and log-rank tests

To examine clinical relevance of Ajuba expression in PDAC tissue, we first performed IHC staining assay on tissue microarray chips (TMA) containing 80 cases of paired resected PDAC and peri-tumor tissue. Of those patients, 48 cases are males and 37 cases are females, and the median age is 62 years old ranging from 32 to 85 years old. The staining intensity of Ajuba on TMA was scored from weak to strong as described in the method, and representative images were shown (Fig. 1a, b). Fifty-seven out of the 80 PDAC samples were scored strong, and 23 cases were scored as weak, while for the peri-tumor samples 28 cases were scored as strong and 52 cases were scored as weak. Statistical analysis indicated that high staining level of Ajuba was strongly associated with PDAC, but was not related to age or gender. Notably, late TNM stage and large tumor volume of PDAC samples showed stronger staining of Ajuba (Fig. 1c, *P* < 0.05). Additionally, PDAC patients with high expression level of Ajuba showed poor survival rate (Fig. 1d, *P* < 0.001). Taken together, these observations suggest that Ajuba could be a marker for PDAC diagnosis and prognosis.

### SP1-mediated gene transcription network is positively correlated with Ajuba expression in multiple cancer types

**Fig. 2:**
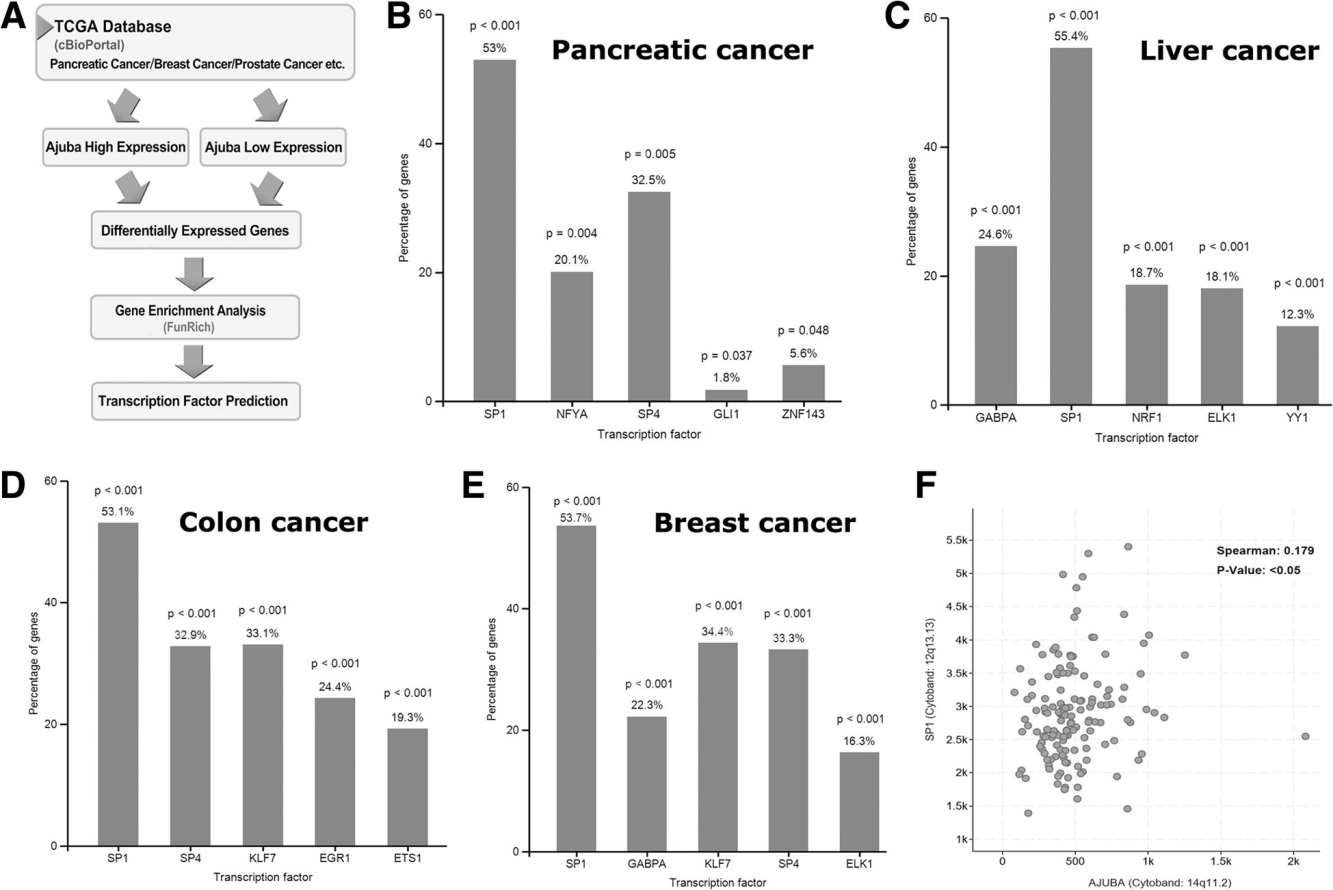
SP1-mediated gene transcription network is positively correlated with Ajuba expression in multiple cancer types. **a** Flow chart shows the process of data analysis and transcription factor prediction. In brief, the dysregulated genes in pancreatic ductal adenocarcinoma (PDAC) tissues with lower or higher Ajuba expression from TCGA database were extracted by cBioPortal (http://www.cbioportal.org), and potential ttranscription factor prediction was analyzed using FunRich software. (**b**, **c**, **d**, **e**) SP1 mediated transcription network is the top hit in PDAC, liver cancer, colon cancer and breast cancer, respectively. **f** the expression of Ajuba and SP1 is positively correlated in PDAC

To identify dysregulated signaling pathways in PDAC tissue along with expression of Ajuba, we performed extensive bioinformatic analysis of the TCGA databases through cBioPortal tool (Fig. 2a). Differentially expressed genes were identified in PDAC tissue with high or low expression of Ajuba and were further analyzed using the tool of Gene Enrichment Analysis (RunRich) to predict the corresponding transcription factors. Notably, 53% of the dysregulated genes were SP1 targets (P < 0.001) (Fig. 2b). Similarly, we extended this analysis strategy to other types of cancers and found that SP1 was the leading transcription factors associated with Ajuba in cancers of liver, colon and breast (Fig. 2c, d, e). We further performed linear regression and spearman correlation analysis of Ajuba and SP1 expression in PDAC tissue in TCGA database, and found that the expression Ajuba and SP1 were positively correlated (Fig. 2f, *P* < 0.05). Collectively, these observations suggest that Ajuba may participate in SP1-mediated gene transcription.

### High expression of SP1 is positively associated with Ajuba in PDAC tissue and predicts poor survival

**Fig. 3:**
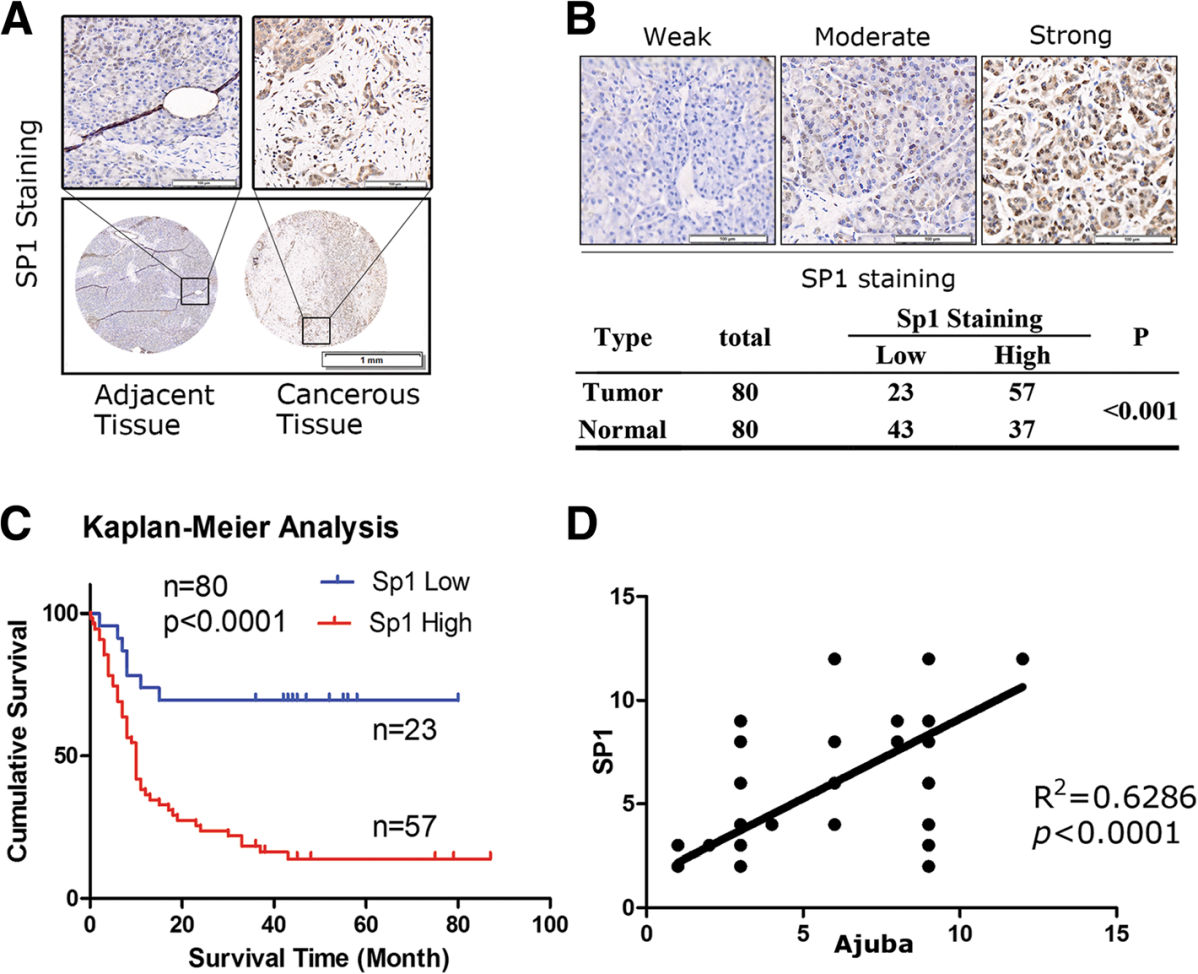
High expression of SP1 predicts poor survival of PDAC. **a** Expression pattern of Ajuba in paired human PDAC specimens (cancerous vs. adjacent tissue) based on intensity and percentage. **b** Representative IHC images of SP1 shows weak, moderate and Strong expression of SP1 (**c**) High expression of SP1 is associated with poor survival of PDAC patients. **d** the protein expression of Ajuba and SP1 is positively correlated in PDAC

To further confirm the correlated expression pattern of Ajuba and SP1 in PDAC tissue, we performed IHC staining assays with antibody specifically against SP1 on the serial TMA chips as used on Ajuba staining (Fig. 3a). Of the 80 paired PDAC tissue, 57 cases showed high expression of SP1, and 23 cases showed low expression; while in the para-tumor samples, 37 cases showed high expression of SP1, and 43 cases showed low expression (Fig. 3b). Statistical analysis indicated that high expression of SP1 was positively associated with PDAC (Fig. 3b), and predicted poor survival of PDAC patients (Fig. 3c). Consistent to the observations obtained from the TCGA database, Ajuba and SP1 protein levels in PDAC tissue were positively correlated (Fig. 3d).

### Depletion of Ajuba markedly dampens SP1-mediated growth of pancreatic cancer cells

**Fig. 4:**
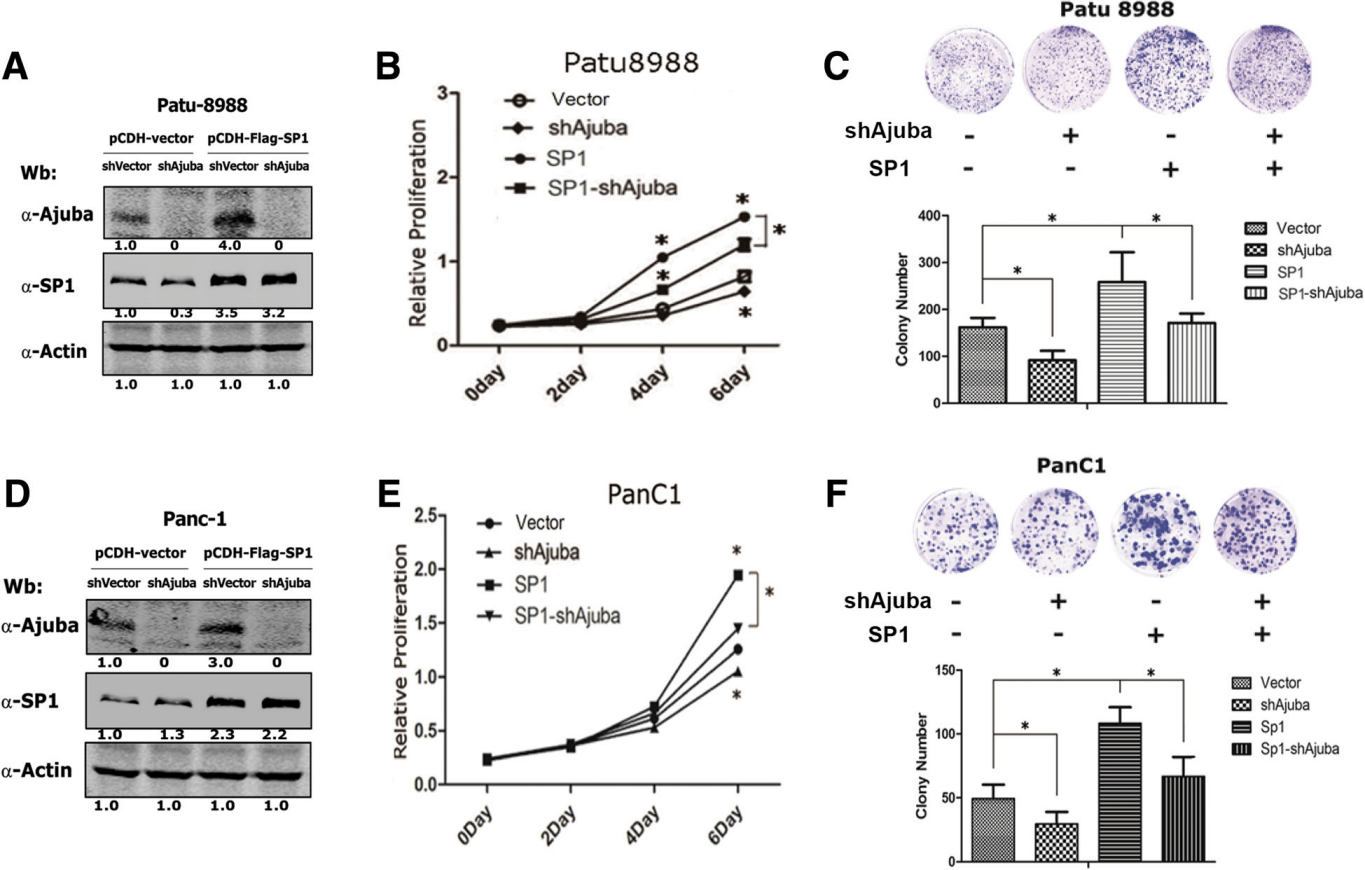
Depletion of Ajuba dampens SP1 promoted cell proliferation of pancreatic cells. **a** Western blot assays showed the expression level of Ajuba and SP1 in Patu 8988 cells. **b** Cell growth rate was detected by CCK-8 assays. **c** Colony formation assays showed the effect of SP1 and Ajuba on the growth of Patu 8988 cells. Colonies were counted and analyzed after 10 days of post seeding. **d** Western blot assays showed the expression level of Ajuba and SP1 in PanC1 cells. **e** Cell growth rate was detected by CCK-8 assays. **f** Colony formation assays showed the the effect of SP1 and Ajuba on the growth of PanC1 cells. Experiments were repeated twice in triplicates. Data shown was from one reprehensive experiment in triplicates

To examine the effect of Ajuba on SP1-mediated cell growth, we established serial cell lines in pancreatic Patu8988 cells stably expressing SP1, shAjuba, SP1/shAjuba and empty vector and the growth of the resulting cells were analysed using methods of CCK8 kit and colony formation assays. The expression of Ajuba and SP1 was first examined by western blot assays showing Ajuba was efficiently depleted and SP1 protein was readily increased (Fig. 4a). Consistent to prior reports, ectopic expression of SP1 markedly increased Patu8988 cell growth, while Patu8988-shAjuba cells displayed the lowest growth rate comparing to the vector control cells. Notably, depletion of Ajuba in Patu8988-SP1 cells significantly dampened the cells growth ability (Fig. 4b). Similar results were observed in colony formation assays: Patu8988-SP1 cells formed the most number of colonies, while depletion of Ajuba reduced the number of colonies (Fig. 4c). In parallel, we performed the same assays in PanC1 cells, and obtained similar results as observed in Patu8988 cells (Fig. 4d, e, f). Collectively, these data strongly suggest that Ajuba may be a regulator of SP1 and is essential for SP1-mediated biological functions.

### Ajuba interacts with SP1 via its LIM domain

**Fig. 5:**
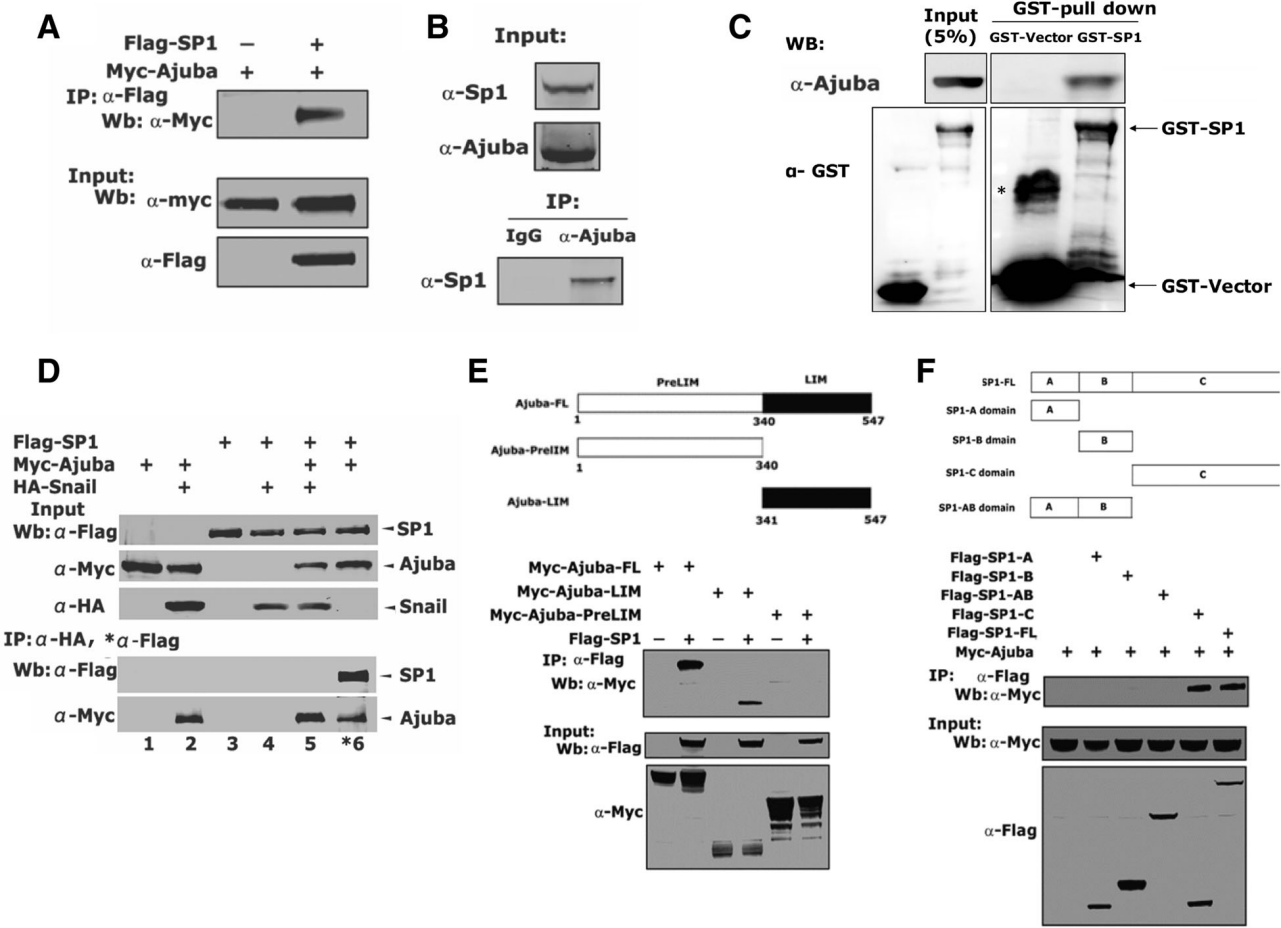
The LIM region of Ajuba binds to the C-terminal SP1 independent of Snail. **a** Ajuba interacts with SP1. Lysates from HEK-293 T cells transiently expressing Myc-Ajuba and/or Flag-SP1 were incubated with Flag M2 beads, and the co-eluted proteins were blotted with Myc antibody. **b** Ajuba interacts with SP1 at endogenous level. Lysates from PanC1 cells were incubated with anti-Ajuba, and the co-eluted proteins were examined using SP1 antibody. Ten percent of total lysates was loaded as Inputs. **c** GST-SP1 and His-Ajuba was respectively expressed in *E. coli* BL21, and in vitro binding assay was performed. *non-specific band. **d** Ajuba/SP1 complex is independent of Snail. Myc-Ajuba, Flag-SP1 and HA-Snail were expressed alone or in combination in 293 T cells and co-IP assays were carried using anti-HA or anti-Flag antibodies. **e** The LIM domain of Ajuba interacted with Sp1. Lysates prepared from HEK-293 T cells transfected with plasmids encoding Myc-Ajuba and its truncation mutants, together with Flag-SP1 were incubated with anti-Flag M2 beads, and co-eluted proteins were examined by anti-Myc. **f** C terminal SP1 interacted with Sp1. C terminus of Sp1 contains a conserved DNA binding domain

To examine if there was an interaction between Ajuba and SP1, we transiently co-expressed Myc-Ajuba and Flag-SP1 in 293 T cells and performed co-IP assays using Flag antibody. Indeed, SP1 readily immunoprecipitated Myc-tagged Ajuba (Fig. 5a). This interaction was further confirmed at the endogenous proteins in Panc1 cells using the antibody specifically against Ajuba (Fig. 5b). To test if their interaction is direct, we expressed SP1 and Ajuba protein in *E. coli* respectively, and in vitro binding assays showed GST fused SP1 could readily pulldown His-tagged Ajuba protein (Fig. 5c). We previously showed Ajuba interacted with Snail and acted as an obligate compressor to repress gene transcription. To examine if Ajuba/SP1 complex was independent of Snail, we co-expressed Flag-SP1, Myc-Ajuba and HA-Snail alone or in combination in 293 T cells and performed co-IP assays Consistently, Snail robustly co-eluted with Ajuba, but failed to co-immunoprecipitated SP1 regardless of the presence of Ajuba (Fig. 5d, lane 6).

To determine the region in Ajuba molecule that interacts with SP1, we made plasmids encoding preLIM or LIM regions respectively and were co-expressed in 293 T cells. co-IP assays showed that SP1 bound to the LIM region, not the preLIM region (Fig. 5e). Moreover, we made serial truncations of SP1, and performed co-IP assays. Notably, Ajuba specifically bound to the C-terminus of SP1containing the DNA binding motif (Fig. 5f). Collectively, these data demonstrate that Ajuba may be a cofactor of SP1 to regulate gene transcription.

### Ajuba functions as a coactivator to enhance SP1 target gene expression

**Fig. 6:**
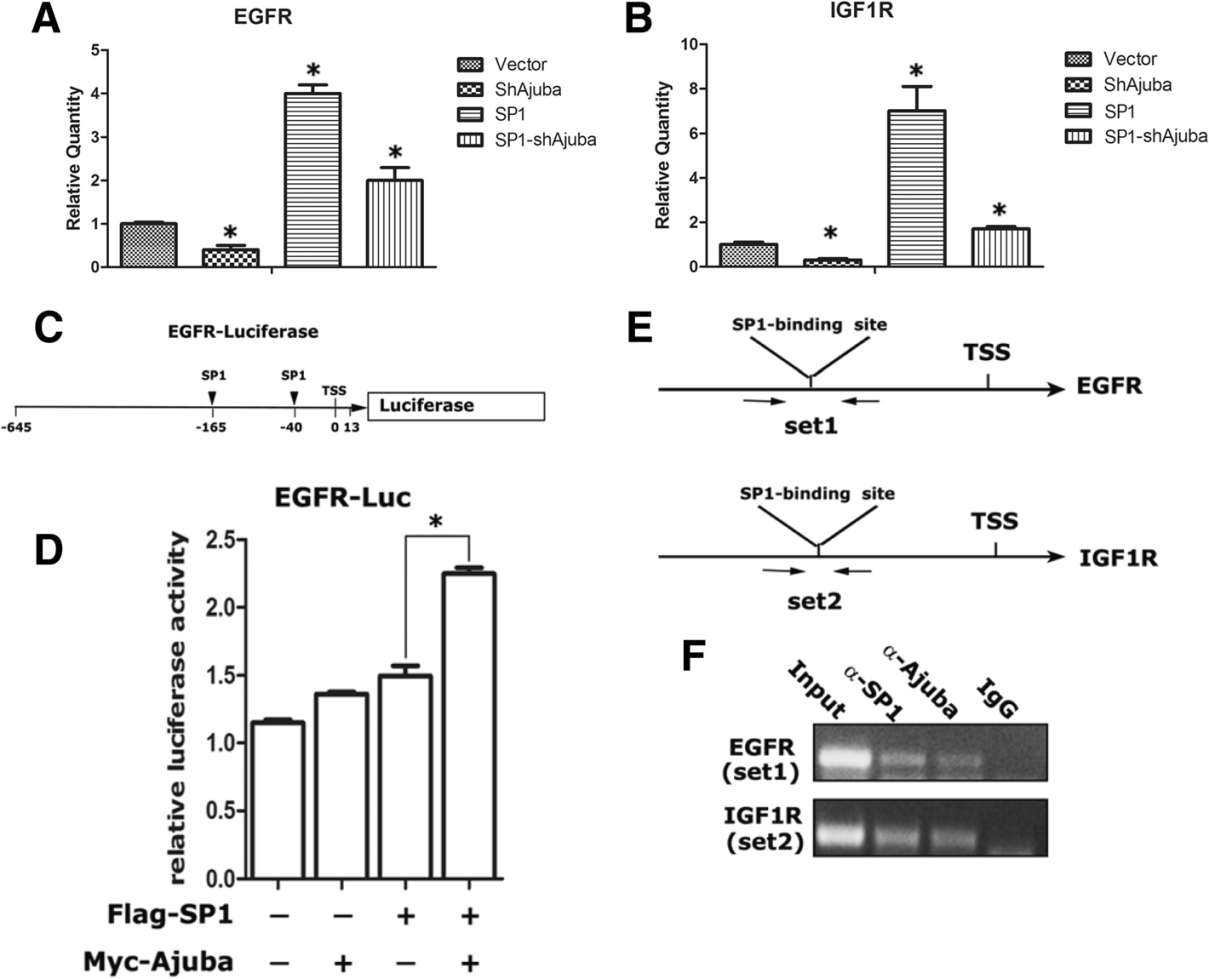
Ajuba is required for SP1 to maximally transactivate target gene expression. **a**, **b** The mRNA level of EGFR and IGF1R was examined in PanC1 cells stably expressing SP1 and/or shAjuba by qRT-PCR assays. **c** Diagram showed the EGFR promoter containing two known functional SP1 responsive elements was cloned into the pGL3 vector to make an EGFR-Luc reporter. **d** Co-expression of Ajuba and SP1 maximally induced EGFR-Luc activity. EGFR-Luc reporters, together with plasmids encoding Flag-SP1 and Myc-Ajuba were transfected into HEK-293 T cells and EGFR-Luc reporter activity was normalized to β-galactose activity. **e** Diagrams showed the primer pairs used for PCR assays for amplification of EGFR and IGF1R promoters flanking functional SP1 responsive elements. **f** ChIP assays showed binding activity of Ajuba and SP1 to EGFR and IGF1R promoter in Panc-1 cells. ChIP assays were performed using SP1 and Ajuba antibodies, and the co-eluted DNA fragments were analyzed by using PCR

To determine the role of Ajuba in SP1-mediated transcriptional regulation, we examined the mRNA level of the best known SP1target genes EGFR and IGF1R in pancreatic cancer Patu8988 cells used for cell growth assays by using qRT-PCR approach. Depletion of Ajuba resulted in low expression of, while forced expression of SP1 increased the expression of EGFR and IGF1R to 4 fold and 7 fold compared to that of the vector control cells (Fig. 6a, b). Notably, depletion of Ajuba in Patu8988-SP1 cells markedly reduced the expression of EGFR and IGF1R (Fig. 6a, b).

To further determine the role of Ajuba in SP1-mediated transcriptional activity, we made an EGFR promoter luciferase reporter containing functional SP1 response elements (Fig. 6c) and performed luciferase assays in PanC1 cells. Expression of Ajuba or SP1 alone only weakly increased the luciferase activity, while in combination of Ajuba and SP1 significantly increased the luciferase activity (Fig. 6d). Next, we performed ChIP assays to examine the binding activity of Ajuba to endogenous SP1 target promoters in PanC1 cells and the enriched DNA fragments were analyzed by qPCR assays using primer pairs flanking the functional SP1 binding sites of the EGFR and IGF1R gene promoters. The data showed that both Ajuba nad SP1 could bind the endogenous promoters of EGFR and IGF1R genes containing the known SP1 binding sites (Fig. 6e). Collectively, these results demonstrate that Ajuba could be a co-activator for SP1 and is essential for induction of SP1 target gene expression.

### The proximal promoter of Ajuba contains tandem SP1 binding sites and Ajuba is induced by SP1 in pancreatic cancer cells

**Fig. 7:**
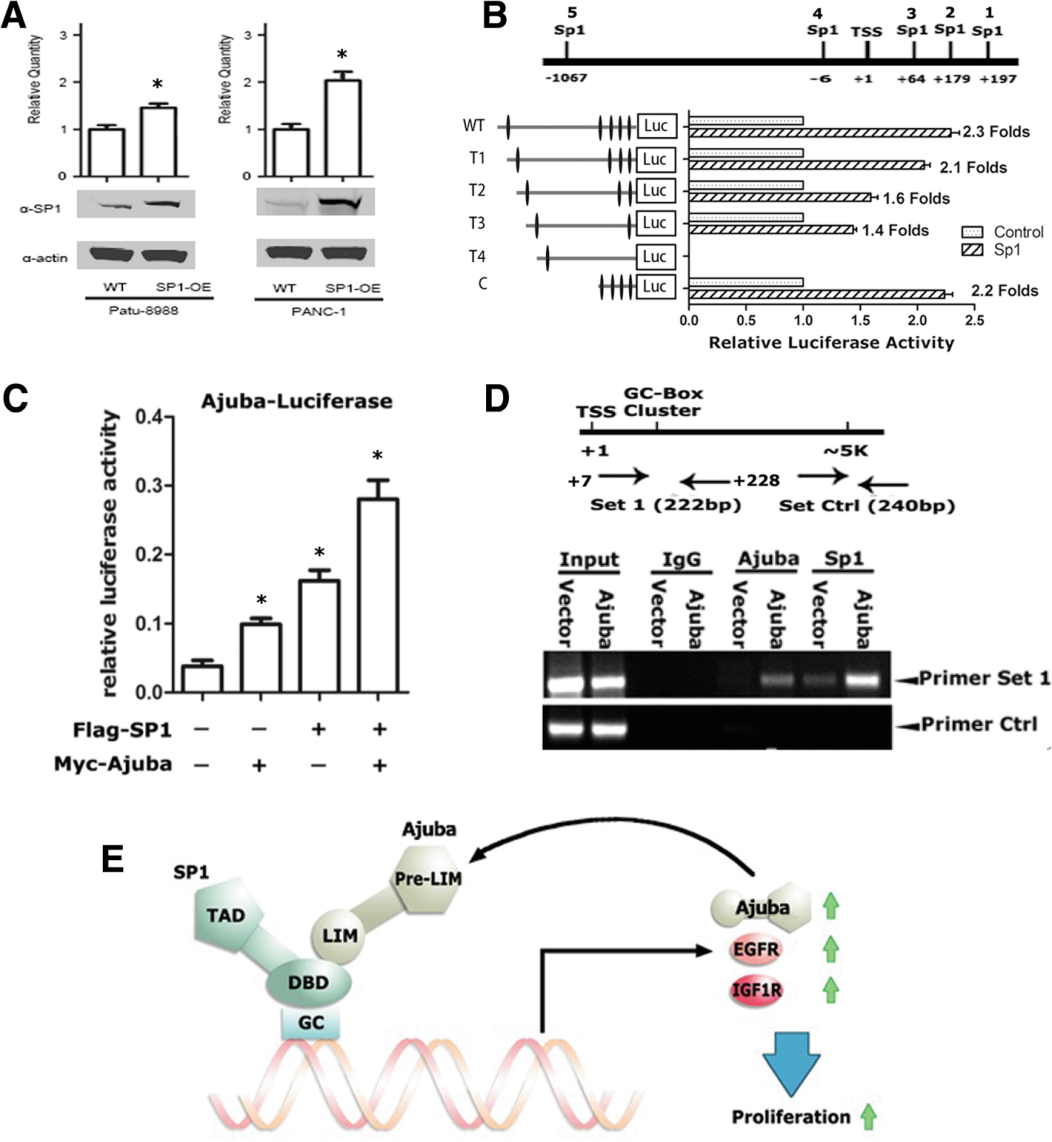
Ajuba promoter contains tandem SP1 responsive elements and itself is a SP1 target gene. **a** SP1 induced Ajuba transcription in Patu8988 and PanC1 cells. The cells were the same as that used in Fig. 5. mRNA level of Ajuba was examined using qRT-PCR. **b** SP1 induces Ajuba promoter activity in PanC1 cells. The Ajuba promoter region contains five predicted SP1 responsive elements, in which the first four binding sites are close to the TSS and form a SP1 binding site cluster. Serial Ajuba promoter deletions were cloned into pGL3 vector and the reporter assays were performed in PanC1 cells. T1-Luc: Deletion of the 1st SP1 binding site. T2-Luc: Deletion of the 1st and 2nd SP1 binding sites. T3-Luc: Deletion of the 1st,2nd and 3rd SP1 binding sites. T4-Luc: Only retain the binding site of the 5th SP1 binding sites. C-Luc: Deletion of the 5th SP1 binding site. **c** Ajuba and SP1 maximally induced full length Ajuba-promoter Luc activity. **d** ChIP assays showed binding activity of Ajuba and SP1 to Ajuba promoter in PanC1 cells, and overexpression of Ajuba enhanced SP1 binding. ChIP assays were performed using SP1 and Ajuba antibodies, and the co-eluted DNA fragments were analyzed by using PCR. **e** Model for Ajuba in SP1 mediated gene transactivation. Ajuba functions as a co-activator of SP1 and itself is a target gene of SP1. Thus, Ajuba/SP1 complex could form a feed forward loop to drive the gene expression and promote cell proliferation

The positive correlation of the expression of Ajuba and SP1 in PDAC tissue prompted us to examine if Ajuba is regulated by SP1. We first examined the expression of Ajuba in Patu8988 and PanC1 cells stably expressing SP1 using qRT-PCR assays, and found SP1 could induced Ajuba expression in both cell lines (Fig. 7a). Next, we analyzed the promoter sequence of Ajuba and found that the proximal promoter of Ajuba contained 4 potential SP1 binding sites. We cloned a series of Ajuba promoter truncations to make various luciferase reporters (Fig. 7b, upper panel), and the luciferase reporter assays were performed in PanC1 cells. The results showed that deletion of the first SP1 site (T1-Luc) and the fifth SP1 site (C-Luc) did not apparently affect the promoter activity, while additional deletion of SP1 sites simultaneously (T2-Luc and T3-Luc) reduced the luciferase activity to 1.6 and 1.4 fold respectively. Remarkably, deletion of all four SP1 binding sites (T4-Luc) resulted in drastically reduction of the transcriptional activity. This data suggests that the tandem SP1 sites in the proximal region of the Ajuba promoter are critical for both basal and SP1 induced activities.

We next examined the effect of SP1 on Ajuba-Luc reporter activity in PanC1 cells, and found SP1 and Ajuba alone could induce the Ajuba-Luc activity, while co-expression of Ajuba and SP1 induced the strongest luciferase activity (Fig. 7c). To determine the binding activities of SP1 and Ajuba on the endogenous Ajuba promoter, we further performed ChIP assays in PanC1-Ajuba and vector control cells using antibodies specifically against SP1 or Ajuba, and the enriched DNA fragments were examined by semi-quantitative PCR. In PanC1-vector cells, both SP1 and Ajuba showed weak binding at the Ajuba promoter containing the tandem SP1 binding sites, while in PanC1-Ajuba cells, both Ajuba and SP1 were readily detected at the Ajuba promoter (Fig. 7d). Taken together, these data indicate that Ajuba itself is a target gene of SP1, and can be induced by SP1/Ajuba complex (Fig. 7e).

## Discussion

Ajuba is a versatile scaffold protein participated in assembly of variety of protein complexes and displays multifaceted biological functions. In the nucleus, Ajuba forms complexes with numerous transcription factors to directly regulate gene transcription [29–31]. Depending on binding to specific transcription factors, Ajuba either acts as a co-repressor by recruiting repressive complexes such as Prmt5, Hdacs and Ezh2, or as a co-activator by recruiting transactivating complexes such as p300/CBP [10, 11, 29]. In this paper, we discover that Ajuba functions as a co-activator of SP1 to induce its target gene, and more interestingly, we identify that Ajuba itself is a target gene of SP1. Ajuba contains functional tandem SP1 responsive elements in its proximal promoter and can be induced by Ajuba/SP1complex. Deletion of all four SP1 binding sites (The T4-Luc) resulted in dramatically reduction of the basal luciferase activity and the SP1 responsiveness (Fig. 7b). In general, SP1 binds to the GC rich elements in its target promoters and is critical for maintaining high level of the target gene transcription. For examples, Sp1 is the key transcription factor to maintain high level of the basal transcription activity of DRG2 and LRP5 promoter [30, 31].

Since Ajuba was first identified as Grb2 interacting protein to enhance MAP kinase activity, various Ajuba interacting proteins have been discovered [32, 33]. In cytoplasm, Ajuba directly interacts with Rac, α-catenin and F-actin to maintain cell-cell adhesion and regulate cytoskeleton assembly; Ajuba also interacts with many signaling transducers to modulate the signal propagation from cytoplasm to nucleus such as JAK/STAT, Hippo, WNT/β-catenin. In nucleus, Ajuba can regulate gene expression by associating with transcriptional factors. Ajuba recruits 14–3-3 and Prmt5 to Snail and mediates the inhibition of E-cadherin expression; Ajuba also interacts with various types of nuclear hormone receptors and functions as an atypical co-regulator. These Ajuba interacting proteins were identified by employing methods of yeast two hybrid system, immunoprecipitation coupled with mass spectrometry, and computer-based motif prediction [10–12, 32]. Here, we provide another strategy to identify Ajuba interacting proteins. By analysing the dysregulated signaling pathways in PDAC tissue along with expression of Ajuba utilizing the public databases such as TCGA databases through cBioPortal (website: http://www.cbioportal.org), the differentially expressed genes were identified in PDAC tissue with high or low expression of Ajuba and were further analyzed using the tool of Gene Enrichment Analysis (RunRich) to predict the corresponding transcription factors. Indeed, SP1 was successfully identified and validated. This strategy can be extended to other transcription factors/co-regulators.

SP1 belongs to the SP / Kruppel-like factor (KLF) transcription factor family, and binds the GC boxes to control transcription of a large pool of developmentally important genes. In many type of cancer tissue, SP1-mediated transcriptional activated is enhanced, which leads to tumorigenesis, progression and metastasis [17–19].

As such, many co-factors have been identified to interact with SP1. Here, we identify Ajuba and SP1 are both highly expressed in PDAC tissue and are positively correlated, and biologically, Ajuba is essential for SP1 promoted cell growth. These findings suggest that Ajuba and SP1 might be biomarkers for PDAC diagnostics, prognosis and targets for new therapeutics.

## Conclusions

Ajuba is a newly defined transcriptional co-regulator and plays important role in various cancers development, while SP1 is a classic transcription factor, and is closely related with a variety of gene expression regulation and cancer development including PDAC. Ajuba functions as a co-activator of SP1 to induce its target genes, and that Ajuba itself is a target gene of SP1. Ajuba/SP1 complex could form a feed forward loop to drive SP1 target gene transcription and promote cell proliferation of pancreatic cancer cells. Clinically, Ajuba and SP1 might be biomarkers for PDAC diagnostics, prognosis and targets for new therapeutics.

## Abbreviations

ChIP: chromatin immunoprecipitation
co-IP: co-immunoprecipitation
IHC: immunohistochemistry
PDAC: Pancreatic ductal adenocarcinoma
RunRich: Gene Enrichment Analysis
TMA: tissue microarray chips
